# Ish: SIMD and GPU accelerated local and semi-global alignment as a CLI filtering tool

**DOI:** 10.1093/bioadv/vbaf292

**Published:** 2025-11-17

**Authors:** Seth Stadick

**Affiliations:** Life Sciences Group, Bio-Rad Laboratories, Hercules, CA 94547, United States

## Abstract

**Motivation:**

To date, there has been no command line utility for performing index-free alignment-based filtering of records. Since filtering using command line tools is a staple of Bioinformatics, this leaves a gap in command line workflows.

**Results:**

Ish is the first composable unix-style command line tool for filtering the input target records to only those that match the input query with a threshold alignment score, using a selectable alignment algorithm and selectable record type. The core alignment algorithms for ish meet or exceed the performance of their reference implementations in Parasail for both SIMD and GPU alignment, as measured by gigacell updates per second (GCUPs).

**Availability and implementation:**

The source code and documentation are available at https://github.com/BioRadOpenSource/ish under the Apache-2.0 License and open for community contributions. Ish is installable with Conda and supports Linux and macOS.

## 1 Introduction

To date, there are few index-free approximate match CLI tools, and none that act explicitly as a filter. There are numerous highly specialized tools in the field of bioinformatics to perform alignment on different sequence types, but all require the preparation of reference sequences into a bespoke index format ahead of time and/or produce output records that are different than the input. In addition, they are usually specific to either protein or nucleotide alignments. Outside of bioinformatics, there are even fewer tools available. The most notable tool is agrep [from TRE (Laurikari 2023)], an approximate matching regular expression implementation that can allow for up to *n* mismatches and specifying mismatch, insertion, and deletion scores. TRE is single-threaded and does not support common record formats in bioinformatics (FASTA, FASTQ, SAM).

Ish addresses this gap in the ecosystem by providing a flexible command-line interface for highly optimized full dynamic programming (DP) matrix alignment algorithms, allowing users to perform alignments between their query and target records, outputting only records that exceed a given alignment score threshold. Ish uses exact alignment algorithms with adaptive gap scoring (affine costs), specifically, local alignment and semi-global alignment. The implementation uses the striped SIMD algorithm described by Farrar (Farrar 2007), and refined by SSW (Zhao *et al.* 2013), with Parasail (Daily 2016) as the reference point, with some modifications.

## 2 Install

Ish can be installed with Conda directly or with any package manager that works with Conda packages such as Pixi.

conda install\

 -cconda-forge\

 -chttps://repo.prefix.dev/modular-community\

 -chttps://conda.modular.com/max\

 ish

## 3 Usage

Ish is designed as a Unix-style stream filter, operating on the principle of text-in, text-out for seamless integration into command pipelines. It outputs only records meeting the alignment threshold. When invoked interactively (attached TTY), matched regions are highlighted and line-based records include terminal hyperlinks to source locations; in pipeline contexts, records are output unmodified to preserve downstream compatibility. See Section 3.1 for further details on selectable record types.

A common use case for ish is searching for a subsequence in a set of sequencing reads. Ish abstracts over both FASTA and FASTQ records and will automatically detect and handle compressed inputs.

  $ishCACTTGGGCCTCAGCTTTCAGAGCCCTCGGG\

  —scoring-matrixactgn\

  —record-typefastx\

  threshold 0.8\

 reads.fa.gz

> contig1

CGGACTCTGGG< highlight >CACTTGGGCCTCAGCAAACAGAGCCCTCGGG</highlight >AGGATCCGGCT

A more general purpose use case for ish is searching for code snippets in a code base. Since ish is alignment-based, it can tolerate misspellings or capitalization differences without additional configuration. By default, ish will recursively search the directory in which it is run, or the directories which it is passed. It can also be given specific files to search.

 $ishsimd_widthof./ishlib

 ./ishlib/matcher/striped_semi_global_matcher.mojo:3: from sys.info import < highlight >simd_width_of</highlight >

 ./ishlib/matcher/__init__.mojo :14: This will override the ‘ < highlight >simd_width_of</highlight > ‘ if the env var ISH_SIMD_TARGET=baseline

### 3.1 Selectable record type

Ish is a command line tool developed with bioinformatics applications in mind but is broadly applicable for approximate matching uses across disciplines. Version 1.0 supports FASTA, FASTQ, and Line record types, allowing it to align only against the sequences of interest in those record types. When matching against FASTA or FASTQ records, Ish is able to achieve performance gains over similar tools like agrep because it bypasses header lines. Additionally, being record type aware allows ish to output records in the correct format, so a matched FASTA record will output the header and sequence instead of just the matched line.

Allowing for matching against lines as a record type allows ish to be used in contexts outside of bioinformatics, such as searching a code base, regular text search, etc. By default, ish will recursively search all non-dotfile non-binary files in the target directory, and output matched lines with the filepath and line number formatted correctly as terminal links if it is attached to a TTY.

### 3.2 Selectable alignment method

To facilitate matching homologous sequences, splice site detection, conserved domain detection, etc., we implemented local alignment (Koerkamp 2025). Semi-global was selected because it generalizes to most other forms of alignment by allowing one to specify which combination ends of the query and target you would like to be “free” from a scoring perspective (Koerkamp 2025). By default, ish allows for both ends of the query sequence to be free but allows specifying any combination.

### 3.3 Selectable scoring matrices

Scoring matrices play an important role in producing high-quality alignments by weighting substitutions according to biological or chemical similarity rather than treating all mismatches equally. For instance, in proteins, substituting similar amino acids (e.g. leucine for isoleucine, both hydrophobic) incurs a smaller penalty than replacing leucine with a charged residue like aspartic acid. Ish supports three scoring schemes: ACTGN for nucleotides (including ambiguous bases), BLOSUM62 (Henikoff and Henikoff 1992) for proteins (derived from observed substitution frequencies in homologous sequences), and ASCII for general text where all mismatches are weighted uniformly.

In addition to alphabet-specific scoring matrices, ish supports affine gap penalties across all alignment methods, further improving alignments by allowing them to represent biology more closely, in the case of protein and nucleotide sequences (Altschul and Erickson 1986).

### 3.4 Scoring threshold

Ish filters based on a configurable scoring threshold. The score is the alignment score found divided by the optimal score for the selected scoring matrix and the gap-open, gap-extend penalty. This is more intuitive than setting a fixed alignment score and is more flexible as a default value when the queries are not known ahead of time. For example, instead of calculating the optimal alignment score for the query and then determining how low a score is tolerable, a threshold score of 0.8 can be specified, which would mean that the alignment score found must be ≥0.8·optimalscore.

## 4 Results

All SIMD benchmarks were performed on AWS EC2 instances: c7a.xlarge (AMD EPYC 9R14 with AVX-512, 3.7 GHz) for x86_64 and c8g.xlarge (ARM Neoverse-V2 with SVE2) for aarch64. GPU benchmarks used g6-series instances with NVIDIA L4 GPUs (7424 CUDA cores, 23 GiB GDDR6, compute capability 8.9).

### 4.1 Striped SIMD alignment

The following benchmarks reproduce a subset of those performed by Daily (Daily 2016), using the UniProt Knowledgebase Release 11.0 (uniprot_sprot.fasta). 31 sequences were used as query sequences ranging in length from 24 to 5478 residues. These were chosen as a subset of the query sequences used by Daily. See the benchmarking utilities in the source code for more details. Each query sequence was aligned against all 549 832 sequences in the database, which range in length from 2 to 35 213 residues, with a median length of 292. The ish striped SIMD implementations were compared against Parasail’s using Parasail’s aligner tool that is meant for benchmarking. A similar aligner tool was created for ish that benchmarks just the alignment method and output generation.

All alignment results were validated against Parasail’s outputs to ensure correctness. The speed is reported in billion (giga) cell updates per second (GCUPS), where a cell refers to a coordinate in the n×m DP matrix. Following convention, GCUPS is normalized by *nm* rather than the total number of values computed across all DP layers. The loading time and profile creation time were excluded from these results, and all results in this section are for a single thread. Only the Blosum62 (Henikoff and Henikoff 1992) scoring matrix was used, since that is what Daily used for comparison between tools. Unlike the original Parasail manuscript, a gap open of 3 and a gap extend of 1 was used, which are the defaults in the SSW tool (Zhao *et al.* 2013). Parasail v2.6.2 was used for all testing and was compiled from source for each machine following the build procedure in the README.

### 4.2 Local alignment evaluation

**Figure 1.**
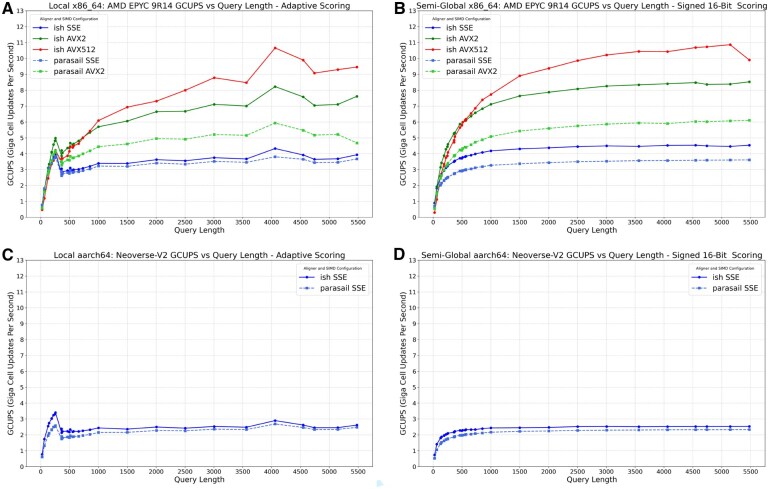
Striped SIMD alignment performance comparison local alignment with adaptive scoring (A, C) and semi-global alignment with signed 16 bit scoring (B, D) compared between Parasail (dashed lines) and ish (solid lines) in protein alignments. (A, B) x86_64. (C, D) aarch64. Different SIMD widths shown as different colors. Ish matches or exceeds Parasail performance across all configurations, with notable improvements on long queries using larger vector sizes.

Figure 1 (left) shows the performance of Parasail and ish over the full range of query sequence lengths using the adaptive scoring mechanism, which is the most common for local alignment. The adaptive mechanism will begin by using a single unsigned 8 bit integer to hold scores, and when an overflow is detected, switch to an unsigned 16 bit integer [see SSW documentation for further discussion (Zhao *et al.* 2013)].

The performance of ish is in line with or slightly improved when compared to the standard set by Parasail. Notably, the larger vector sizes usable by ish perform better on long query sequences than the shorter vector sizes in Parasail. On x86_64, ish can take advantage of AVX512 instructions when the hardware supports them, which are slower at short lengths but outstrip AVX2 with queries of approximately length 750 or greater.

### 4.3 Semi-global alignment evaluation

Figure 1 (right) shows the performance of Parasail and ish across the full range of query sequence lengths using signed 16 bit integers for scoring. Byte size scoring was skipped since signed 8 bit integers overflow quickly with semi-global alignment scoring, twice as fast as unsigned 8 bit integers with local alignment. The performance of ish is again equal to or greater than the standard set by Parasail.

### 4.4 GPU alignment

The approach taken for doing GPU based alignment is intentionally naive and runs a scalar version of semi-global alignment [from Parasail (Daily 2016)] on N threads of the GPU, with a definable coarse-graining factor. When multiple GPUs are detected, the work will split between them.

### 4.5 GPU alignment evaluation

**Figure 2.**
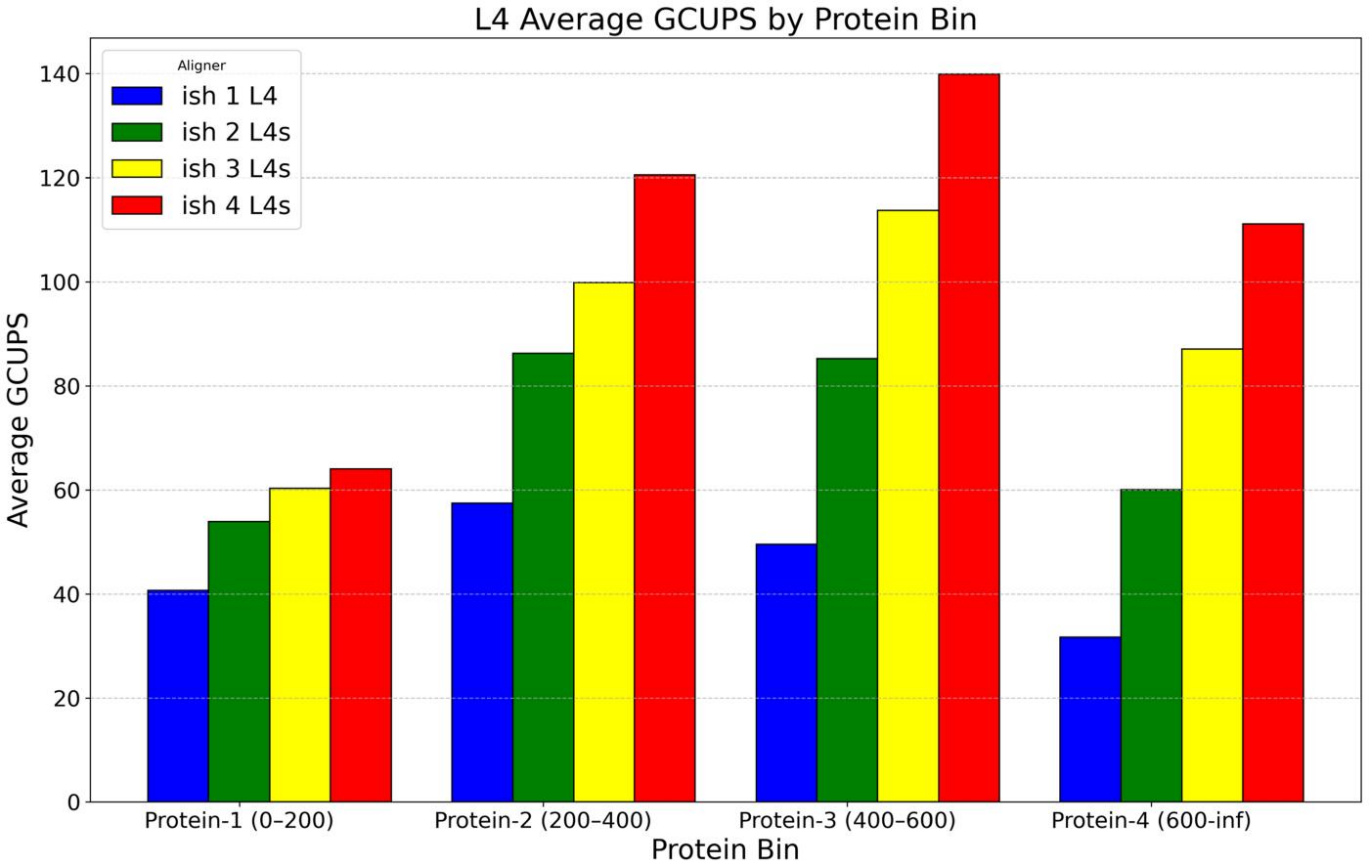
GPU based semi-global alignment performance semi-global alignment performance in GCUPs binned by query length. Each color represents the number of GPUs used, demonstrating ish’s ability to scale with multiple GPUs.

Figure 2 illustrates the raw performance of the GPU kernel used in ish. Specifically, it measures the GCUPs starting from the start of the data copy to the GPU, and stopping after the transfer off the GPU. Input sequences are sorted by length and per-GPU. The same benchmarking tool was used for both GPU and SIMD alignments, structured after the Parasail aligner.

Although our algorithm performs reasonably fast, it is still more efficient to use the CPU on smaller inputs. On inputs where the target file size is roughly 4GB or larger the overhead of setting up the GPU buffers becomes worthwhile.

### 4.6 CLI Tool comparisons

For all comparisons, the same reference data as used in the parasail (Daily 2016) was used. The input query was P01111 (length 189 residues), or the first 50 residues of P01111 when the length is truncated.

The only CLI tool that works like ish, by fuzzy matching input records and filtering them based on a score, is agrep (Laurikari 2023). agrep is a grep-like tool that uses the TRE regex engine, which in turn allows for regexes to match even when there are a pre-selected number of “errors.” It works on line-based records by default but does allow for defining a custom record delimiter regex. This does not work well with multi-line FASTA or FASTQ records because it will not match across the newlines, and will needlessly try to match against the header lines. ish supports line records and also supports a generic ASCII scoring matrix.

To compare accurately to agrep, ish was run in line mode with ascii scoring using a query that matches a single line of a multiline FASTA. Additionally, the scoring threshold of 0.75 for ish was translated for agrep, allowing up to 12 of the 50 characters to be errors. The same scoring of -2 for mismatch and -3 for insertions and deletions was used for both tools.

Ish dramatically outperforms agrep in every context. Ish is 3× faster running single threaded, scales with the number of threads, and when using both threads and GPU is 75× faster. Additionally, the ability to search FASTA records further speeds up ish, as seen in Table 1 where ish is 92× faster.

**Table 1.** CLI tool performance comparison.^a^

Comparison	Tool	Configuration	Time (s)
*Line Record search (query length 50)*			
	agrep	1 thread	371.5
	ish	1 thread	124.6
	ish	24 threads	9.5
	ish	2 GPUs	4.9
*FASTA Record search (query length 50)*			
	agrep	1 thread (still lines only)	371.5
	ish	1 thread	81.4
	ish	24 threads	7.2
	ish	2 GPUs	4.1
*Semi-global FASTA (query length 189)*			
	glsearch36	24 threads	6.8
	ish	24 threads	5.5
	ish	3 GPUs	4.9

a ish outperforms agrep by 75× (with GPUs) and glsearch36 by 1.4×. FASTA-aware parsing provides additional 1.5× speedup over line-based search. Ish defaults to using semi-global alignments with the query ends free.

Glsearch is part of the fasta36 suite of tools (Pearson 2016), and was chosen for comparison because it implements the same underlying alignment algorithm as ish, albeit with a more of a focus on report generation than unix-style streaming. As seen in Table 1, ish is 1.38× faster in the best case, and even when only using the CPU dataflow, it is still 1.26× faster.

Agrep version 0.9.0 was used and built out of the TRE repository (Laurikari 2023). glsearch version 36.3.8i as part of the fasta36 distribution (Pearson 2020). Mojo v25.6.0 was used for ish v1.4.0.

## 5 Discussion

Ish fills a niche that no other tool currently occupies; fuzzy matching on the CLI that is record type aware and uses a full alignment algorithm. This is a significant step forward from agrep (Laurikari 2023) in terms of performance and a large step forward from fasta36 (Pearson 2016) in terms of ergonomics. Sassy is another recent addition in this category that is blazingly fast. Sassy does not act as a stream filtering tool, and it implements a fundamentally different alignment algorithm. For further comparison between ish and sassy, see Beeloo and Koerkamp (2025).

Ish will be useful to a wide audience for approximate matching against ASCII text. For bioinformatics, ish has a role in the development process, checking outputs for specific sequences, and possibly even as a filtering tool in pipelines to help filter input reads for specific splice sites or other unique features.

Ish’s alignment algorithm implementations in Mojo were a success. Performance was greater than or equal to the reference implementation, and the CLI tools performance is competitive with other similar tools.

These results demonstrate not only the immediate utility of ish, but also highlight Mojo’s potential as a platform for rapidly developing high-performance, portable sequence tools that bridge the gap between CLI usability and modern hardware acceleration.

## Data Availability

*The data underlying this article are available in* UniProtat https://ftp.uniprot.org/pub/databases/uniprot/previous_releases/release-2015_11/knowledgebase/knowledgebase2015_11.tar.gz as well as specific sequences that are available in https://github.com/jeffdaily/parasail/tree/fb985ee4f2302c72c87d7d56443712b2d920b106/data.
